# Recognition of Tumor-Associated Antigens and Immune Subtypes in Glioma for mRNA Vaccine Development

**DOI:** 10.3389/fimmu.2021.738435

**Published:** 2021-09-17

**Authors:** Shuai Ma, Yixu Ba, Hang Ji, Fang Wang, Jianyang Du, Shaoshan Hu

**Affiliations:** ^1^Department of Neurosurgery, The Second Affiliated Hospital of Harbin Medical University, Harbin, China; ^2^Translational Medicine Research and Cooperation Center of Northern China, Heilongjiang Academy of Medical Sciences, Harbin, China; ^3^Department of Neurosurgery, Shandong Provincial Hospital Affiliated to Shandong First Medical University, Jinan, China; ^4^Department of Neurosurgery, Emergency Medicine Center, Zhejiang Provincial People's Hospital Affiliated to Hangzhou Medical College, Hangzhou, China

**Keywords:** mRNA vaccine, immune subtype, glioma, immune landscape, tumor antigens

## Abstract

**Background:**

Although mRNA vaccines have been efficient for combating a variety of tumors, their effectiveness against glioma remains unclear. There is growing evidence that immunophenotyping can reflect the comprehensive immune status and microenvironment of the tumor, which correlates closely with treatment response and vaccination potency. The purpose of this research was to screen for effective antigens in glioma that could be used for developing mRNA vaccines and to further differentiate the immune subtypes of glioma to create an selection criteria for suitable patients for vaccination.

**Methods:**

Gene expression profiles and clinical data of 698 glioma samples were extracted from The Cancer Genome Atlas, and RNA_seq data of 1018 glioma samples was gathered from Chinese Glioma Genome Atlas. Gene Expression Profiling Interactive Analysis was used to determine differential expression genes and prognostic markers, cBioPortal software was used to verify gene alterations, and Tumor Immune Estimation Resource was used to investigate the relationships among genes and immune infiltrating cells. Consistency clustering was applied for consistent matrix construction and data aggregation, Gene oncology enrichment was performed for functional annotation, and a graph learning-based dimensionality reduction method was applied to describe the subtypes of immunity.

**Results:**

Four overexpressed and mutated tumor antigens associated with poor prognosis and infiltration of antigen presenting cells were identified in glioma, including TP53, IDH1, C3, and TCF12. Besides, four immune subtypes of glioma (IS1-IS4) and 10 immune gene modules were identified consistently in the TCGA data. The immune subtypes had diverse molecular, cellular, and clinical features. IS1 and IS4 displayed an immune-activating phenotype and were associated with worse survival than the other two subtypes, while IS2 and IS3 had lower levels of tumor immune infiltration. Immunogenic cell death regulators and immune checkpoints were also diversely expressed in the four immune subtypes.

**Conclusion:**

TP53, IDH1, C3, and TCF12 are effective antigens for the development of anti-glioma mRNA vaccines. We found four stable and repeatable immune subtypes of human glioma, the classification of the immune subtypes of glioma may play a crucial role in the predicting mRNA vaccine outcome.

## Introduction

Glioma is a fatal malignancy with a 5-year survivability rate of only 5%. It is ranked in the thirteenth in terms of cancer-related deaths, accounting for 2.5% of global cancer deaths in 2018 (1). Because limited glioma is largely asymptomatic in its initial stages, more than 80% of individuals are usually diagnosed at advanced stages, which diminishes the efficacy of surgical intervention. Besides, patients who undergo complete tumor resection usually experience recurrence within 2 years after surgery (2). Additional treatment approaches, including chemotherapy, molecularly targeted agents, and immune checkpoint inhibitors, have limited impact given inherent chemo- and immune resistance. Recently, tumor vaccines against IDH1 have been successful in slowing the progression of glioblastoma GBM (3). Although these vaccines conferred only a limited time survival benefit, the results are promising enough to expand the potential of vaccines associated with GBM.

Typical tumor vaccines comprise a tumor antigen with/without an adjuvant that reprograms the immune system to recognize and eliminate cancer cells (4). As a result, they offer the benefits of comparative nontoxicity, minimal nonspecific influence, a wide treatment window, and evocation of durable immune memory. Thus, it is possible for tumor vaccines to combat the drug resistance, side effects, restricted therapeutic efficacy, and elevated costs associated with standard chemotherapy and immunotherapy. Depending on the antigenic form, tumor vaccines can be categorized into peptide, dendritic cell (DC), DNA, tumor cell, and RNA vaccines. Peptide vaccines have the advantages of cheap, easy to produce long peptides, Th and CTL epitopes, not HLA-restricted Personalized and semi-personalized peptides, high epitope concentration, and the drawbacks of peptide vaccines are short peptides with no or few epitope cells, limited to selected epitopes/antigens, poor immunogenicity, need adjuvants (5). DNA vaccines have the advantages of not requiring cellular production, intrinsic adjuvant effect, high cost-effectiveness, and direct production. However, there is a potential risk of insertion leading to mutations and the need to enter the nucleus after successful transfection reduces the efficiency of DCs cells (6). DC vaccines measure antigen presentation efficiency and DC cell maturation rates, but it does not have fully mature DC cells, and tumor-compromised DCs may cause tumor tolerance and labor-intensive (7). Tumor cell vaccines are whole-cell vaccines composed of inactivated allogeneic tumor cell lines or autologous tumor cells. They contain characterized and non-characterized tumor antigens. It will contain Th and CTL epitopes, and is also acceptable for allogeneic vaccines. However, it also has poor clinical outcomes and allogeneic HLA rejection vaccines (8). In contrast, momentous technology innovations and study investments have made mRNA a prospective therapeutic tool in the field of vaccine development and protein substitute therapy in the past decade (9). The mRNA vaccine is mainly applied through the following routes. 1, Dendritic cells delivered mRNA vaccines (10, 11), 2, Direct injection mRNA cancer vaccines (12), 3, Lipid nanoparticle (LNP) delivered mRNA cancer vaccines (13). The disadvantages of mRNA vaccines include the need for adjuvants and the rapid rate of extracellular degradation. Compared to other vaccines, the mRNA vaccines have several beneficial features. First, safety: because mRNA is a noninfectious, nonintegrating platform, there are few potential threads of infection or insertional mutations. Besides, mRNA is degraded in natural cellular processes and its half-life *in vivo* can be modulated by using different modifications and delivery approaches. The intrinsic immunogenicity of mRNA can be deregulated to further improve safety. Second, therapeutic effect: various types of modifications make mRNA more stabilized and translatable. By converting mRNA into a vector molecule that allows accelerated uptake and expression in the cytoplasm, efficient *in vivo* delivery can be accomplished. mRNA is the minimal genetic carrier, thus avoiding anti-carrier immunization and allowing repeated mRNA vaccination. Third, manufacture: mRNA vaccines have the promising potential for rapid, promising, and scaled-up production, largely due to the high yield of *in vitro* transcriptional responses (12).

The goal of this study was to investigate new glioma antigens for the development of mRNA vaccines and to map the immune profile of glioma to identify appropriate recipients for vaccination. We confirmed four candidate genes relevant to poor survival and antigen-presenting cell (APC) invasion from a repository of genes with overexpression and mutation in glioma. Based on immune-associated gene clustering, we defined 4 formidable immune subtypes and 10 modules of glioma and verified them in Chinese Glioma Genome Atlas (CGGA) cohort. It corresponds to different clinical, molecular, and cellular signatures for each immune subtype. In conclusion, the immune pattern of glioma was determined by analyzing the distribution of the pertinent gene signatures in individual patients. Our results suggest the existence of a complicated tumor immune microenvironment (TIME) in glioma patients. This research provides a theoretical basis for the development of mRNA vaccines.

## Methods and Materials

### Data Extraction

The standardized gene expression profile (FPKM), as well as clinical data of 698 glioma patients, were gathered from The Cancer Genome Atlas (TCGA, https://www.cancer.gov/tcga), and the RNA_seq and clinical data of 1018 glioma patients were downloaded from the CGGA (http://www.cgga.org.cn/). A total of 2138 immune-related genes were gathered from the ImmPort (https://www.immport.org/shared/home) and InnateDB databases (https://www.innatedb.ca/) and then merged with glioma transcripts (56754) to obtain 1439 immune-related genes.

### GEPIA Analysis

Gene Expression Profiling Interactive Analysis (14) (GEPIA, http://gepia2.cancer-pku.cn, version 2) was used to integrate differentially expression genes (DEGs) and patient prognosis data. The analysis of variance was applied to screen for DEGs with |log2FC| values >2 and Q values <0.01. Overall survival (OS) and relapse-free survival (RFS) were assessed using the Kaplan-Meier method with a median cut-off and compared using the log-rank test. The hazard ratio was calculated using a cox proportional risk regression model. Parameter settings were consistent in each analysis and were not adjusted for any p-value. p <0.05 were regarded as statistically significant.

### cBioPortal Analysis

CBio Cancer Genology Portal (cBioPortal, http://www.cbioportal.org) was employed to incorporate raw RNA-seq data from TCGA and other databases and to compare genetic variations in glioma (530 LGG samples and 604 GBM samples). p < 0.05 was regarded as statistically significant (15). Select the LGG and GBM projects on the cBioPortal website and go to the “Explore selected studies” interface to download all mutated genes separately.

### Immunohistochemical

Immunohistochemical staining was obtained from The Human Protein Atlas (https://www.proteinatlas.org/), and immunohistochemical scores of TP53, IDH1, C3, TCF12 in glioma and normal brain tissue were obtained from the “hpar” package.

### TIMER Analysis

The relationship between tumor immune infiltrating cell abundance and glioma-associated genes was performed and visualized by Tumor Immune Estimation Resource (TIMER, https://cistrome.shinyapps.io/timer/). Modules were obtained by analysis of gene expression, somatic mutations, clinical regression, and somatic copy number alterations. Purity adjustments were chosen using spearman correlation analysis. p- <0.05 was regarded as statistically significant (16).

### Immune Infiltration Analysis

Single-sample gene set enrichment analysis (ssGSEA) was utilized to characterize the relative infiltration of 28 immune cells in the TME. A panel of characteristic genes for every immune cell type was derived from a recent paper. In ssGSEA, an enrichment fraction is shown for each immune cell type in terms of relative abundance. In the analysis, the ssGSEA scores were normalized to a unit distribution, where 0 is was the minimal value for each immune cell type with 1 being the maximum value for each immune cell type (17).

### Generation of Immunescore, Stromalscore, and ESTIMATEScore

The ESTIMATE algorithm estimates the proportion of the immune- stromal component of the TME for each sample by means of an estimation package loaded in R language version 4.0.5, expressed in the form of three scores: Immunescore, Stromalscore and ESTIMATEScore, which are positively correlated with the proportion of immune, substrate and the sum of both, respectively, implying that the higher the respective scores, the greater the proportion of the corresponding components in the TME (18).

### Identification and Verification of the Immune Subtypes

The 1439 immune-related genes were clustered according to expression profiles, and a consensus matrix was structured to define the appropriate immune subtypes and gene modules (19). A segmentation algorithm around Medoid using a “1-Pearson correlation” metric of distance was used, and 500 guided sessions were performed, involving 80% of the patients in the detection cohort. The clustering sets ranged from 2 to 10, and the best partition was determined by assessing the consistency matrix and the consistency accumulative distribution function. The immune subtypes were then confirmed in standalone CGGA cohorts with the same settings. Concordance of immune subtypes among discovery and verification cohorts was quantified by computed pearson correlation among the immune subtypes.

### Prognostic Assessment of Immune Subtypes

The prognostic value of each immune subtype was analyzed by log-rank test, and ANOVA was applied to identify the association of immune subtypes with disparate immune-related molecular and cellular characteristics.

### Analyses of Mutation Immune Subtypes

To reveal relevant genetic alterations, single nucleotide variants (SNV), single nucleotide polymorphisms (SNP), and minor insertions (INS) or deletions (DEL) with default parameters were identified using MuTect2 (http://www.broadinstitute.org/cancer/cga/mutect) based on pairwise comparison files (tumors and matched germline). Somatic mutation and copy number variation (CNV) profiles were obtained from the TCGA data portal (https://portal.gdc.cancer.gov/). Somatic mutation data, which were sorted based on Mutation Annotation Format (MAF), were analyzed using the R package ‘maftools’. Significant of copy number amplifications for decreases were detected using GISTIC 2.0 with a threshold of false discovery rate (FDR) Q < 0.05 (20, 21).

### Establishment of the Immune Landscape

The immune landscape was constructed based on graphical learning for dimensionality reduction analysis, using a normally distributed “Monocle” package of dimensionality reduction functions to provide visualization of the immune subtype distribution among each patient (22). The largest number of components was set at as 4, and the tree discriminant dimensionality reduction method was employed. Finally, the immune landscape was visualized using functional graph cell trajectories with colored coding of immune subtypes.

### WGCNA

Weighted Gene Coexpression Network Analysis (WGCNA) was employed to identify the hub genes among 1439 immune-related genes. First, to make our gene distribution conform to the scale-free network, we established an adjacency matrix according to the connectivity based on an optimal β value and converted the adjacency matrix into a topology overlap matrix (TOM). Then, the dissimilarity between genes was employed to cluster the genes for the TOM we obtained. Finally, recognized TOMs were defined as the factors for hierarchical clustering with dynamic tree-cutting algorithms to discriminate modules, with a size of 25 as the minimum module (23). To further explore the association between clinical parameters and module eigengenes in each module.

## Results

### Confirmation of Potential Antigens of Glioma

**Figure 1:**
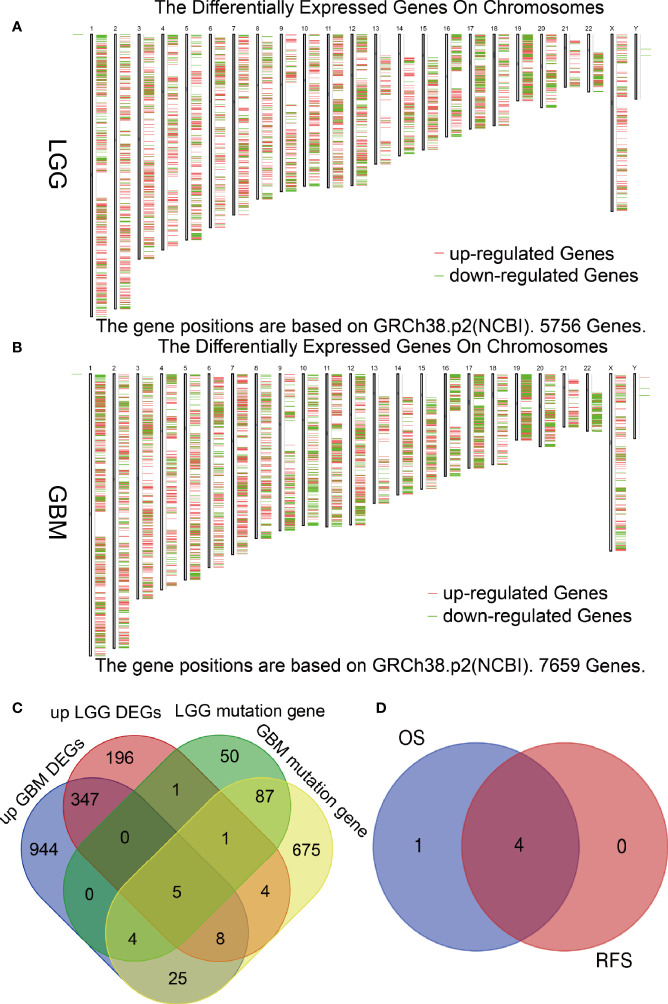
Screening for potential tumor antigens in glioma. **(A, B)** Identification of potentially relevant antigens in glioma. Chromosomal distribution of differentially expressed genes in low-grade glioma (LGG) and glioblastoma (GBM). **(C)** Venn diagram showing the overlapping genes among genes grouped based on up GBM DEGs, genes grouped based on up LGG DEGs, genes grouped based on LGG mutation genes, and genes grouped based on up GBM mutation genes. **(D)** Statistically significance of the 5 hub genes for predicting overall survival and relapse-free survival (p < 0.05).

Tumor antigens are mainly derived from up-regulated genes and mutation genes (24). To confirm the up-regulated DEGs of glioma, we performed differential analysis of LGG and GBM by GEPIA and obtained 5756 and 7659 differentially expressed genes (|logFC|>1, P<0.05), respectively. There were 3982 up-regulated DEGs in LGG and 5221 up-regulated DEGs in GBM, potentially encoding tumor-associated antigens (**Figures 1A, B**). To confirm the mutation genes of glioma, the 4736 and 8182 mutated genes was from the cBioPortal website, which potentially encoding tumor-specific antigens were screened by analyzing the genomic fraction and mutation counts altered in individual LGG and GBM samples. We selected the genes with logFC>2 and mutation rate >1% to obtain 1366 (GBM) and 563 (LGG) DEGs (**Table S1**) and 809 (GBM) and 148 (LGG) mutated genes (**Table S2**). Finally, we used the Venn Diagram web tools and obtained 5 hub genes among the genes based on up LGG DEGs, the genes based on up GBM DEGs, the genes based on GBM mutation genes, and the genes based on LGG mutation genes (**Figure 1C**). Subsequently, tumor antigens associated with prognosis were screened from the above genes as candidate genes for the development of mRNA vaccines. Five genes were strongly correlated with OS in glioma patients, of which 4 genes were significantly associated with RFS (**Figure 1D**). These results revealed that 4 hub genes have considerable potential for mRNA vaccine development.

### Confirmation of Tumor Antigens Relevant to Glioma Prognosis and Antigen Presenting Cells

**Figure 2:**
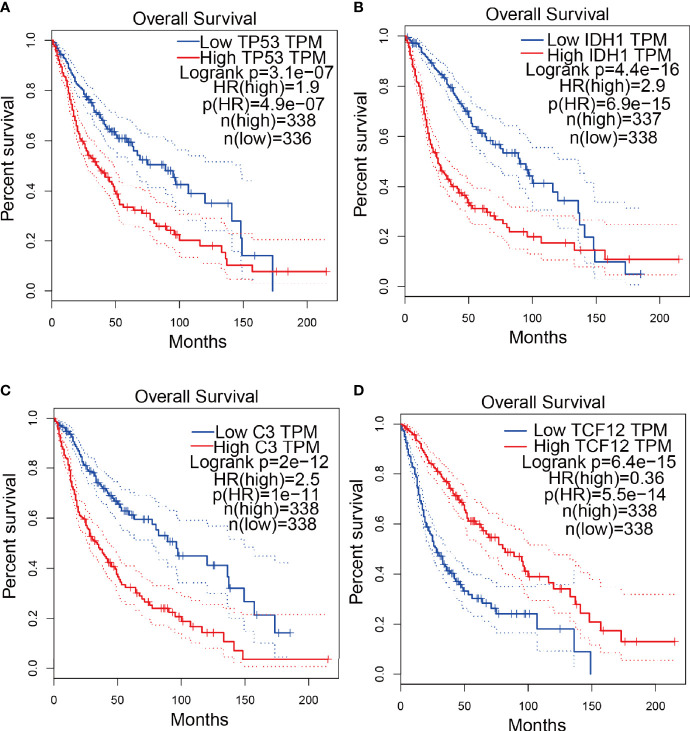
Confirmation of tumor antigens in glioma. **(A-D)** KM survival curves for glioma patients grouped based on median gene expression values **(A)** TP53, **(B)** IDH1, **(C)** C3, **(D)** TCF12.

**Figure 3:**
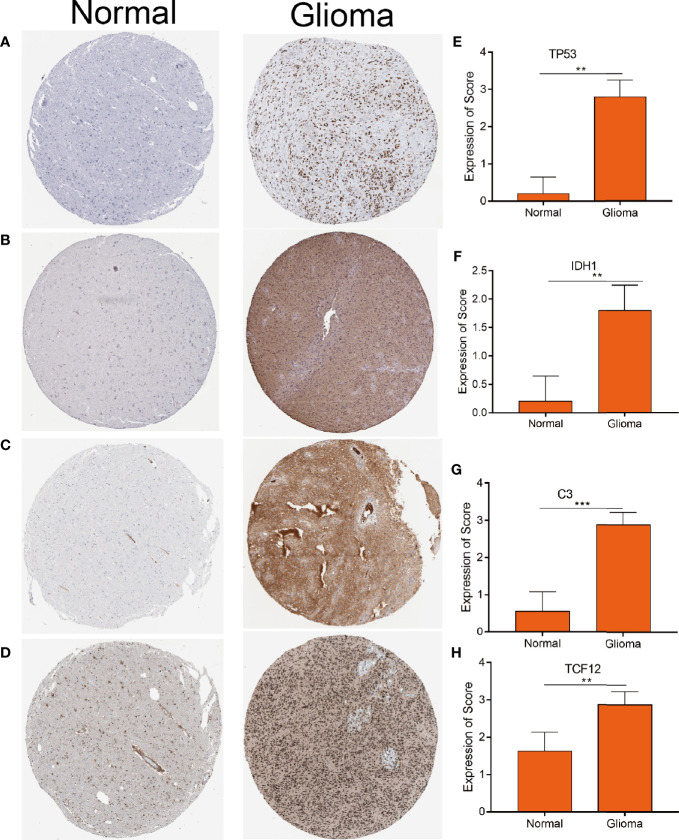
Distinctively expressed proteins in human glioma tissue and normal brain tissue. **(A–D)** The protein expression of TP53 **(A)** IDH1 **(B)** C3 **(C)** and TCF12 **(D)**. Representative photos are shown (100×), Scale bar = 100 μm. **(E–H)** The protein expression scores of glioma tissue and normal brain tissue are shown. ***p < 0.001 by t-test indicated a significant difference from normal tissues. **p < 0.01.

To explore the correlation between the 4 potential antigens and patient survival, we further investigated their relationship with survival. Patients who overexpressed TP53 (the famous tumor suppressor gene) in tumors had remarkably shorter survival than those with low TP53 expression. Likewise, overexpression of IDH1, C3, and TCF12 was also associated with OS (**Figures 2A-D**). We then performed timeROC curves validation of the 4 hub genes, and the results showed that the AUC of C3, TCF12, and IDH1 genes were all greater than 0.6, and the AUC of TP53 was greater than 0.5, further indicating that these four genes have an important role in the prognosis of glioma (**Figures S1A-D**). In summary, a total of four candidate genes for glioma development and progression were identified. Furthermore, high expression levels of IDH1 and C3 were obviously linked to enhanced tumor infiltration by macrophages, DCs, and/or B cells (**Figures S2B, D**). TP53 and TCF12 also showed a trend toward increased immune cell infiltration, although their expression levels were more varied (**Figures S2A, C**). These discoveries demonstrate that certain tumor antigens may be directly targeted and presented to T cells by APCs and recognized by B cells to invoke an immune response, and thus are hopeful candidates for the development of anti-glioma mRNA vaccines. We further verified the protein levels of the four hub genes by immunohistochemistry, and the results showed that TP53, IDH1, C3, and TCF12 were significantly more highly expressed in glioma than in neuronal cells (**Figures 3A–H**), and this result was consistent with the expression of mRNA, further demonstrating the potential of applying these four genes as mRNA vaccines.

### Establishment of Latent Immune Subtypes of Glioma

**Figure 4:**
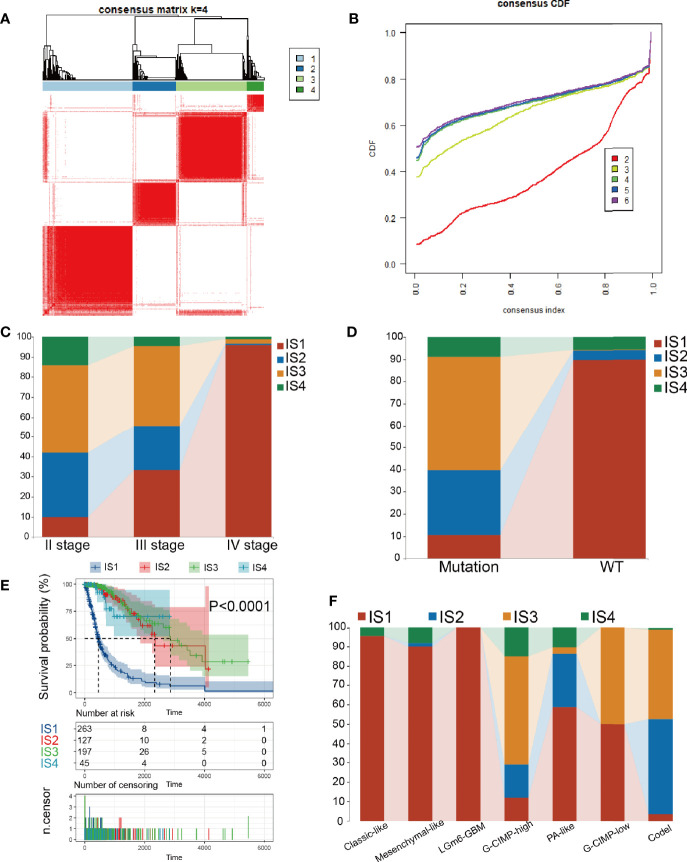
Consensus clustering in TCGA. **(A)** Color-coded heatmaps corresponding to the consensus matrices. **(B)** Consensus Cumulative Distribution Function (CDF) Plot for immune-related genes in glioma. **(C, D)** Distribution of IS1-IS4 between WHO stages and IDH1 types in the TCGA cohort. **(E)** Kaplan-Meier curves survival based on the 4 glioma immune subtypes in the TCGA cohort. **(F)** Distribution of IS1-IS4 between methylation types in the TCGA cohort.

Immune subtypes can be employed to reflect the immune state and microenvironment of the tumor (25), which can help recognize patients who are likely to benefit from vaccination. Hence, we investigated the expression profiles of 1439 immune-related genes in 698 glioma samples from the TCGA database and 1018 samples from the CGGA database to structure consensus clusters. Dependent on their accumulative distribution functions as well as functional incremental areas, we selected k=4 for stable clustering of immune-related genes (**Figures 4A, B** and **Table S3**) and obtained four immune subtypes named IS1-IS4. The distribution of diverse tumor stages and grades within the immune subtypes showed that patients diagnosed with different stages were regularly clustered (**Figure 4C**). The increasing proportion of samples in IS1 with increasing grade, indicated that the immune subtypes were very meaningful in the diagnosis of glioma disease progression. Similar results were observed for the immune subgroups in the clustering based on IDH1 mutation status (**Figure 4D**). Based on the seven subgroups categorized based on the multi-omics data (G-CIMP-low, G-CIMP-high, codel, classic-like, mesenchymal-like, LGm6-GBM, and PA-like), we found that IS1 and IS3 samples had the highest proportion (**Figure 4F**). Then, we analyzed the prognosis of the 4 immune subtypes and found that IS2, IS3, and IS4 had a better prognosis, while IS1 had the worst prognosis (**Figure 4E**). The immune-related gene expression profiles were validated in the CGGA database using the same approach, and samples were clustered into 4 immune subtypes (**Figures S3A, B** and **Table S4**). Consistent with the outcome of the TCGA cohort analysis, the immune subtypes also had significant prognostic differences in the CGGA cohort (**Figure S3D**). IS1, IS2, and IS3 dominated the immunophenotyping clustering in different grades of gliomas (**Figure S3C**). Taken together, these results suggest that immune subtypes can be applied to predict the prognosis of glioma patients with better precision than conventional graded staging, as validated in the CGGA cohort.

### Relevance Between Glioma Immune Subtypes and Immune Modulators

**Figure 5:**
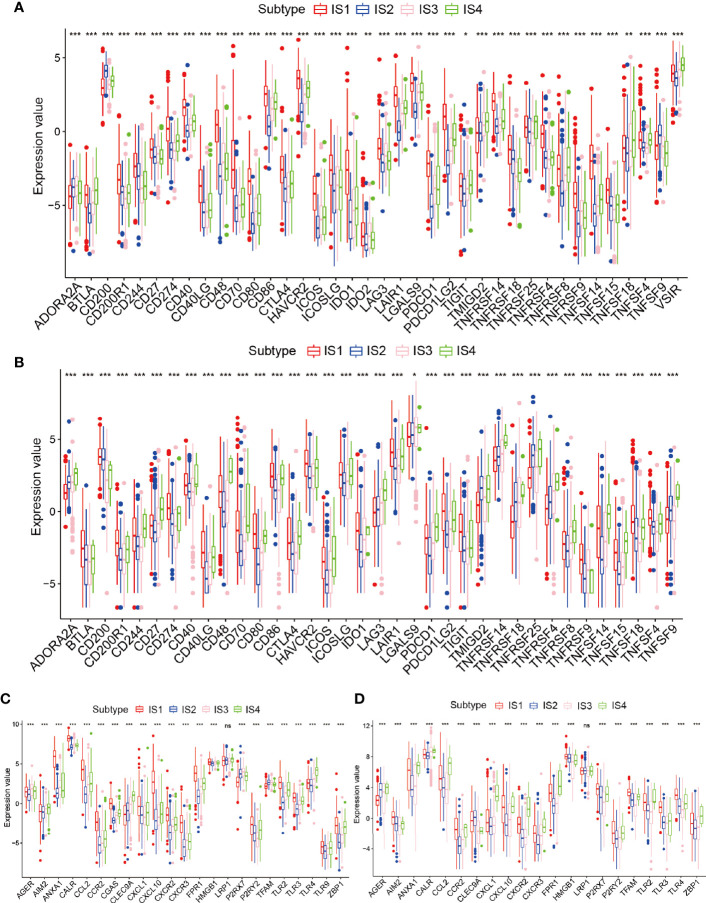
Relationships among the immune subtypes and ICPs and ICD modulators. **(A, B)** Distribution of ICP genes among the glioma immune subtypes in the TCGA and CGGA cohorts. **(C, D)** Distribution of ICD genes between the glioma immune subtypes in the TCGA and CGGA cohorts. *p < 0.05, **p < 0.01, ***p < 0.001 and ns, non-significant.

Because of the importance of immune checkpoints (ICPs) and immunogenic cell death (ICD) regulators in cancer immunization, we next performed an analysis of ICPs expression levels in diverse subtypes (26, 27). Forty ICP-related genes were detectable in the TCGA cohort. For example, CD200R1, CD244, CD274, CD27, CD40, CD48, CD40LG, CD70, CD80, CTLA4, CD86, HAVCR2, ICOS, ICOSLG, IDO1, LAIR1, LAG3, LGALS9, PDCD1, PDCDILG2, TNFRSF14, TNFRSF18, TNFRSF9, TNFRSF14, TNFRSF4, and TNFRSF15 were upregulated in the IS1 immune type in the TCGA cohort (**Figure 5A** and **Table S5**). Besides, the overexpressed ICPs in the CGGA cohort were also concentrated in the IS1 immune type in the CGGA cohort, and the overall expression levels were consistent with those of the ICPs in the TCGA cohort (**Figure 5B**). Furthermore, 23 ICD genes were tested in the TCGA cohort, 22 of which (95.6%) were significantly different between immune subtypes. For example, AGER, AIM2, CLEC9A, P2RX7, TFAM, TLR4, and TLR9 were significantly increased in IS3 and IS4 in the TCGA cohort (**Figure 5C**). Besides, 19 ICD genes were detectable in the CGGA cohort, 18 of which (94.7%) showed the same pattern in the TCGA cohort. For instance, AGER, CALR, CCL2, CXCL1, CXCL10, CXCR2, CXCR3, FPR1, HMGB1, LRP1, TLR2, and ZBP1 were upregulated in IS4 in the CGGA cohort (**Figure 5D**). Collectively, immune subgroups can indicate the expression levels of ICD regulators and ICPs and may serve as potential biomarkers for mRNA vaccines. mRNA vaccines are less effective in patients with high ICPs expression and more effective in patients with upregulated ICD regulators.

### Cellular and Molecular Characteristics of the Immune Subtypes

**Figure 6:**
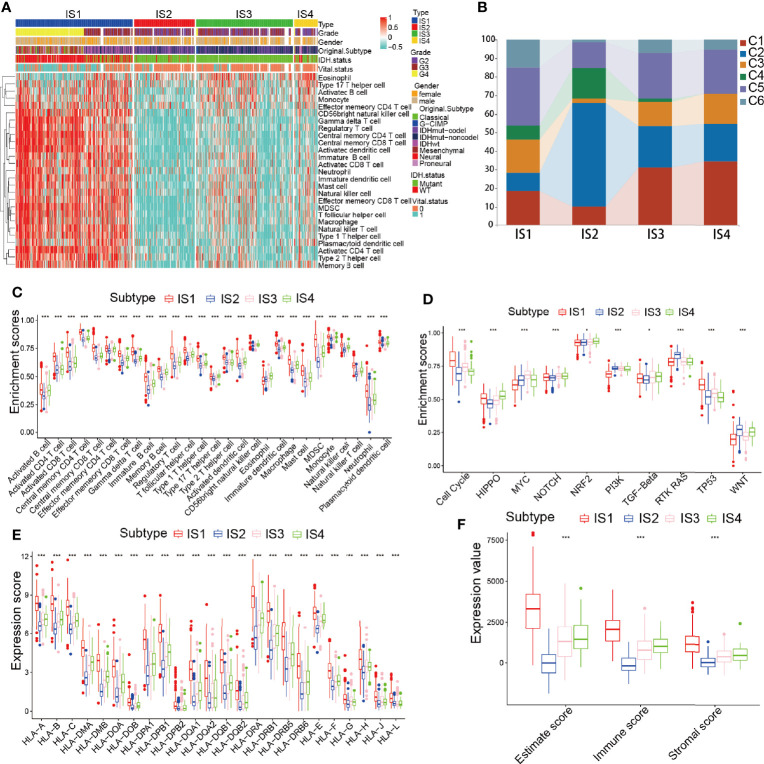
Immune types of immune cell infiltration in the TCGA cohort. **(A)** Heatmap analysis of the distinct enrichment scores of 28 immune cells among the glioma immune subtypes. **(B)** Distribution of IS1-IS4 between pan-cancer immune types (C1-C6) in the TCGA cohort. **(C–F)** Distribution of 28 immune cells **(C)**, 10 oncogenic pathways **(D)**, human leukocyte antigen (HLA) molecules **(E)**, and ESTIMATEScore **(F)** across the 4 immune types. *p < 0.05 and ***p < 0.001.

It was shown that the response to mRNA vaccines depends on the immune state of the tumor. Therefore, by using ssGSEA in the TCGA and CGGA cohorts to score previously reported 28 signature genes, we further assessed the immune cell component of the immune subtypes. The immune cell component was divided into 4 clusters. IS1 and IS4 showed analogous immune cell scores in the CGGA and TCGA cohorts (**Figures 6A**, **S4A** and **Tables S6, 7**). From these results we found that GBM and IDH1 mutated patients were mainly concentrated in the IS1 group (**Figure 4D**), illustrating that our subgroups can further complement and respond to the different molecular characteristics of patients. Meanwhile, there were significant differences in immune cell composition between the immune subtypes. For example, IS1 and IS4 had significantly higher scores for eosinophils, activated CD8 T cells, activated B cells, monocytes, and effector memory CD4 T cells than IS2 and IS3, while memory B cells, macrophages, and memory CD4 T cells had higher scores in IS2 and IS3 than in IS1 and IS4 (**Figures 6C** and **S4C**). Thus, IS1 and IS4 had an “active” immune phenotype, while IS2 and IS3 had a “suppressive” immune phenotype. All these findings suggest that immune subtypes may reflect the immune status of glioma and may identify appropriate patients for mRNA vaccination. These antigenic mRNA vaccines can evoke immune infiltration in patients with immunosuppressive IS2 and IS3 tumors. To confirm the stability of these immune types, we next analyzed the relationship between these four immune subtypes and the six reported previously pan-tumor immune subtypes (C1-C6) (25), in which glioma is predominantly located in the C1, C2, and C5 clusters. IS1, IS3, and IS4 predominantly overlapped with C1 and C5, and IS2 overlapped with C2 and C4. The high proportion of C5 and C6 samples in IS1 was in line with the worst prognosis in those clusters, as shown in **Figure 6B**. The long survival time of IS4 patients may be a result of the high proportion of C3 samples in IS4. We enriched the expression of 10 oncogenic pathways in 4 immune subgroups and showed that IS1 was significantly enriched in the 10 pathways, indicating high malignancy of the IS1 group (**Figures 6D** and **S4D**). Given that T cells can only recognize neoantigens “presented” to them by HLA molecules of the immune system, predicting which neoantigens will bind strongly to HLA molecules and be recognized by T cells is a critical step in the preparation of tumor vaccines (28). Hence, we investigated the expression of HLA antigens among different the immune subgroups in the TCGA cohort. HLA antigens were significantly higher in IS1 and IS4 than in IS2 and IS3. In the CGGA cohort, HLA-DMB, HLA-DPB1, HLA-DPA1, HLA-DQA2, HLA-DRA, HLA-DRB1, and HLA-J were significantly higher in IS1 and IS4 than in IS2 and IS3 (**Figures 6E** and **S4B**). Then we investigated the correlation of these 4 hub genes with HLA, and the results showed that IDH1, TCF12, C3 had high correlation with HLA (**Figure S12A**). Finally, we analyzed the immune scores of the four immune subgroups and found that the ESTIMATEScore and Immunescore were higher in IS1 and IS4 than in IS2 and IS3 (**Figure 6F**). This result was consistent with the immune active groups (IS1 and IS4) and the immunosuppressive groups (IS2 and IS3). In summary, immune subtypes can reflect the cellular and molecular characteristics of glioma patients and indicate their immune state. These findings are an important step forward in our understanding of glioma as discrete disease subsets and in the development of appropriate mRNA vaccines based on such discrete characteristics.

### Relationship Between Immune Type and Mutational Status

**Figure 7:**
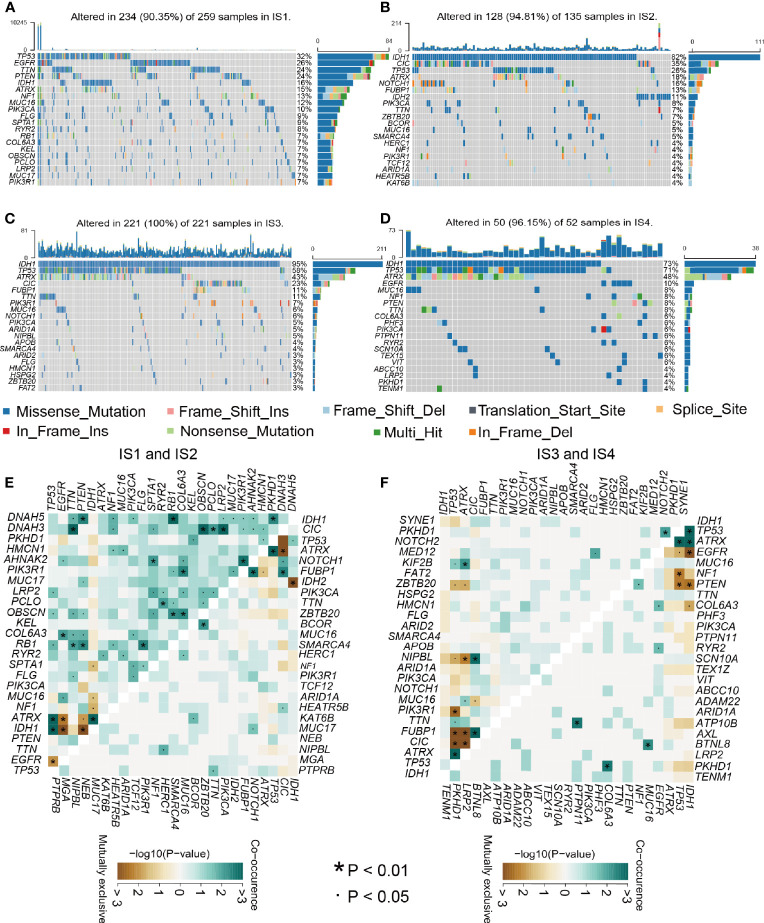
Relationship between mutation rate and immune subtypes. **(A–D)** Waterfall diagram of the four immune subtypes. **(E–F)** Heatmap showing the mutual co-occurrence and exclusive mutations within the top 25 frequently mutated genes.

Relevant literature suggests that the immune status may also be related to mutation (29). Greater tumor mutational burden (TMB) and somatic mutation rates are associated with greater immunity against cancer (30). Therefore, we calculated mutations and TMB for every patient using the TCGA mutation dataset and performed analyses across all immune subtypes. Among the four immune subtypes, IS3 had the highest mutation rate (100%), followed by IS4 (96.15%), IS2 (94.81%), and IS1 (90.5%). The IDH1 mutation rate was the lowest in IS1 at 16% and was 82%, 95%, and 73% in IS2, IS3, and IS4, respectively (**Figures 7A–D**). IDH1 mutation significantly affects the prognosis of glioma patients, so the difference in IDH1 mutation among the immune subtypes may be one of the factors affecting the survival time of patients (31). Interestingly, TP53, IDH1, CIC, and EGFR occupied the top three positions in the four immune types and thus might be potential mRNA vaccine targets, besides there were interactions among them underlying a variety of tumor-related biological processes in glioma, which indicates that they may be primarily concerned with tumor progression. Next, we performed a study of the co-occurrence landscape using the top 25 mutated genes with the comet algorithm. Nine pairs (IDH1-EGFR, ATRX-CIC, TP53-CIC, IDH1-IDH2, FUBP1-TP53, CIC-TP53, FUBP1-ATRX, CIC-ATRX, and EGFR-IDH1) exhibited mutually exclusive mutations compared to the pervasive mutually exclusive landscape, suggesting that they may have redundant effects in the same pathway and a selective advantage of retaining copy of the mutation between them (**Figures 7E, F**). TMB was significantly higher in IS1 and IS4 than in the other two groups of the four immune subgroups (**Figure S5B** and **Table S8**). After inspecting transcriptional alterations in the 4 immune types as described above, we further investigated whether there were differences between the 4 subtypes at the genomic level. Somatic mutations, including SNV, SNP, INS, and DEL, were analyzed and visualized using the R package “maftools”, the SNPs and total in the IS1 and IS4 were also outnumbered by those in IS2 and IS4. While the majority of genomic variants were missense mutations (60%) in the four immune types (**Figures S5A, D** and **Tables S8, 9**). For SNV, all samples from glioma patients were studied, and C>T and T>C were the most common types among the four immune subtypes. For most types of SNV, IS1 had significantly higher levels than the other three immune types (**Figure S5C**). Next, we performed immune grouping based on variant allele frequency and found that IS1 and IS3 showed significantly higher expression than IS2 and IS4. Thus, we can speculate that there is a difference in tumor heterogeneity between IS1, IS3 patients, and IS2, IS4 patients (**Figures S5E–H**). Finally, we performed driver gene analysis for the four immune types, and the results showed that the main driver genes of IS1 were IDH1 and PLCH2 (**Figure S6A**). In contrast, the driver genes of IS2 and IS3 were IDH1, IDH2 (**Figures S6B, C**), and IS4 was IDH1 (**Figure S6D**). After the above analysis, we found that IDH1, IDH2, and PLCH2 may be the main driver genes of glioma, so these three genes are likely to be important targets of mRNA vaccine as well. A large number of copy number variant (CNV) amplification and deletion regions are significantly associated with antioncogene or oncogenes, and CNV of tumor-associated genes can have a significant impact on tumorigenesis and metastasis, affecting patient prognosis (32, 33). IS3 was found to have a significantly higher CNV level than the rest of the immune subgroups (**Figures S7A–D**). The mutated genes in IS1 were mainly distributed in the posterior part of the samples, while those in IS2, IS3, and IS4 were mainly distributed in the anterior part of the samples (**Figures S7E–H**). The findings suggest that immune subtypes could forecast the somatic mutation rates, SNP, INS, SNV, DEL, and TMB of glioma patients. The above finding of our 4 subtypes may pave the way for the subsequent development of mRNA vaccines.

### Immune Landscape of Glioma

The immune landscape of glioma was constructed using immune-related gene expression profiles (**Figure S8A**). The horizontal axis and the vertical coordinate correlate with multiple immune cells except for eosinophils (**Figure S8B** and **Table S10**). In summary, immune subtype-based immune patterns can accurately identify the immune component of each glioma patient and predict their prognosis, which facilitates the selection of personalized treatment regimens targeting mRNA vaccines.

### Confirmation of the Glioma Immune Gene Coexpression Module and Hub Genes

**Figure 8:**
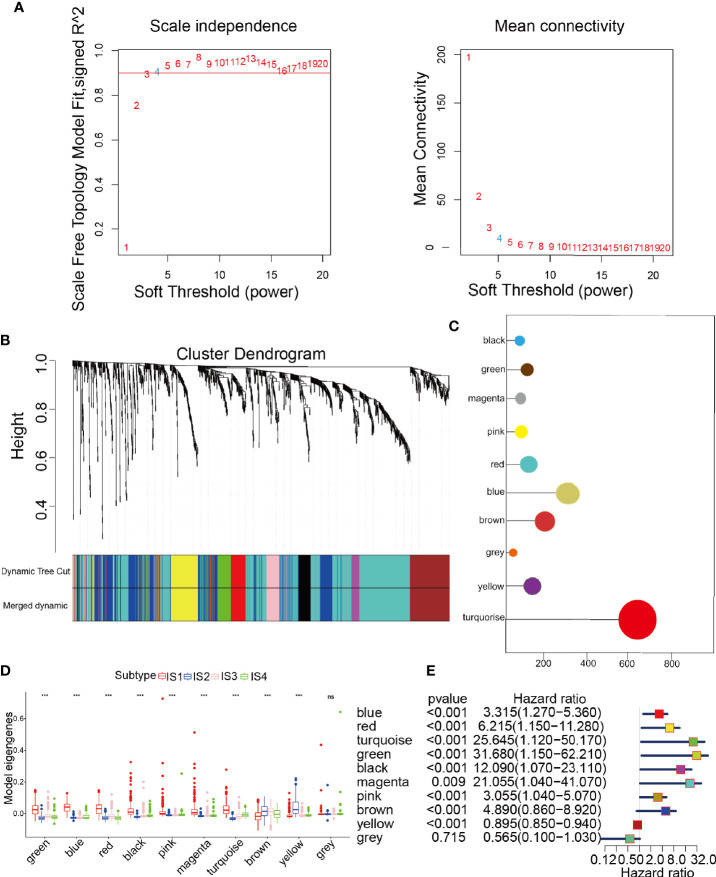
Characterization of the immune gene coexpression module of glioma. **(A)** Determination of the scale-free fit index (β) for every soft threshold power. **(B)** Treemap of all immune-related genes clustered based on the TOM matrix. **(C)** Number of genes in every module. **(D)** Differential distribution of module eigengenes in glioma immune types. **(E)** Univariate Cox regression analysis of nine significant models. ***p < 0.001 and ns, non-significant.

**Figure 9:**
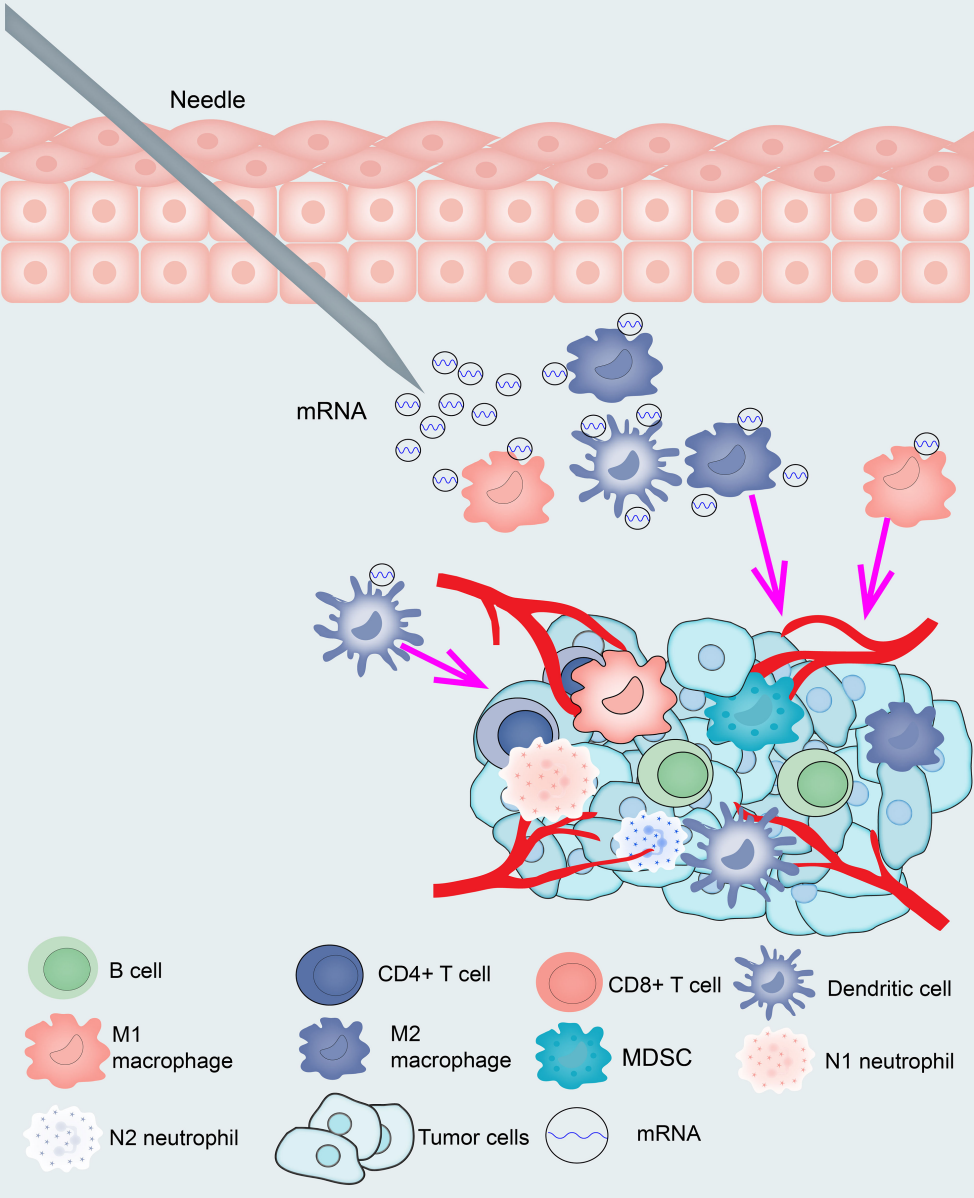
Diagram of mRNA vaccine application pattern in glioma.

The samples were clustered by WGCNA with a soft threshold of 4 in a scale-free network, and 1439 immune-related gene coexpression modules were identified (**Figure 8A**). The proximity and topology matrix were obtained according to the β value, and the topology matrix we obtained was clustered according to the dissimilarity between genes. The gene dendrogram was generated by mean linkage hierarchical clustering. The colored rows on the bottom of the tree diagram show the module assignments determined by dynamic tree cutting (**Figure 8B**). The eigengenes of every module were computed by merging the closed modules into new modules with height = 0.25, deep split = 5, and minimum module size = 25. As shown in **Figure 8B**, A total of 1439 immune-related genes were divided into 10 modules. We further analyzed the gene numbers distribution of the 10 modules (**Figure 8C**). The distribution of the four immune subtypes in the characteristic genes of the ten modules was further analyzed, and significant differences were found in the distribution of nine modules eigengenes (**Figure 8D** and **Table S11**). IS1 exhibited the greatest number of eigengenes in the green, blue, red, black, pink, magenta and turquoise modules, while IS2 showed the lowest number of features in the green, blue, black, pink, magenta, and turquoise modules. The prognostic correlation analysis revealed that the remaining modules, except the gray module, were distinctly relevant to the prognosis of glioma (**Figure 8E**). For the 9 modules of prognosis-related genes, the analysis displayed that superior expression was associated with better prognosis in the TCGA cohort, which was consistent with the above observations (**Figures S9A–I**). Then, we analyzed component 1 and component 2 separately in relation to each of the nine modules (**Figures S10A–O**). The results showed that the blue, magenta, and turquoise modules had correlation values greater than 0.75 and correlated negatively with component 1 and component 2 (**Figures S11A–D**). Moreover, GO enrichment analysis of the blue, magenta, and turquoise modules showed that the blue module was relevant to endothelial cell proliferation, the magenta module was associated with T cell activation, neutrophil migration, and the turquoise module was associated with leukocyte proliferation (**Figures S11E–G**). Infiltration and activation of immune cells in tumor tissue largely affect the therapeutic potential of mRNA vaccines for patients with certain immune subtypes of cancer. Therefore, mRNA vaccines may not be suitable for patients with high expression of genes in the magenta and turquoise modules. Two hub genes with >95% correlation in the magenta and turquoise modules, including GP2 and IFNA16, were ultimately identified as potential mRNA vaccine biomarkers. Finally, **Figure 9** shows the application pattern of mRNA vaccines,which could be helpful for the clinical application of mRNA vaccines in the future.

## Discussion

To the best of our knowledge, this is the first study to select potential glioma antigens for the development of mRNA vaccines. The construction of aberrant expression and the mutational profiles of glioma samples identified a series of targetable antigens, of which TP53, IDH1, C3, and TCF12 are hopeful glioma mRNA vaccine antigens. Upregulation of their expression was associated not only with OS, but also with high infiltration of APCs and B cells. Thus, there are crucial roles for these antigens in the evolution and progression of glioma, and they can be directly processed and presented to CD8 T cells when there is sufficient lymphocyte infiltration to induce immune attack. Although these candidate genes require further functional validation in the future, their potential for mRNA vaccine exploitation is favored by previous reports. The TP53 gene is an oncogene that is mutated in more than 50% of all malignant tumors, dysfunction of this gene seriously affects the cell cycle and induces tumor development, and the high level of TP53 in glioma was found to significantly affect patient survival (34). Mutations in the IDH1 gene are an important molecular markers identified in recent years and are closely associated with the development of gliomas. Patients with IDH1 mutations have a better prognosis than those without IDH1 mutations (35). Recent studies have showed significant progress in the treatment of gliomas with IDH mutations through immunotherapy with vaccination (3). IDH1/2 mutations are ideal tumor-specific antigens because they occur at specific codons in IDH1 and IDH2 and are commonly found in glioma cells (36). C3 is at the confluence of two activation pathways, plays a pivotal role in the activation of the complement system, and is a key molecule in the activation of alternative pathways, participating in immune regulation, for example, by acting as a nonspecific stimulatory signal for B-cell activation, acting as a mitogen for B-cell proliferation, synergizing with antibodies to enhance the effects of glioma and stimulating the release of prostaglandin E (PGE) from monocytes (37, 38). TCF12 is involved in the modulation of transcription from the RNA polymerase II promoter, the immune response, myogenesis, and the modulation of transcription. A recent study identified the presence of TCF12 mutations in oligodendrogliomas that impair TCF12 transcriptional activity and associated with more aggressive tumor types, suggesting that these TCF12 mutations may have an important role in their development (39, 40).

Since mRNA vaccines are effective in only a small proportion of tumor patients, we categorized glioma into four immune subtypes according to immune-related gene expression profiles to identify the population suitable for vaccination (41, 42). Each immune subtype corresponded to distinct clinical, molecular and cellular characteristics. For example, patients in IS1 and IS4 tumors with elevated TMB or somatic mutation rates are likely to be more responsive to mRNA vaccines. After detecting transcriptional alterations as mentioned above, we further surveyed whether there was evidence of differences between immune subtypes at the genomic level. According to the results of mutation types, we found that the total numbers of mutations, SNPs, and SNVs of the IS1 and IS4 groups with immune activation status were significantly higher than those of the IS2 and IS3 groups, suggesting that there is some similarity between immune types and mutation types. Finally, driver gene analysis of the immune types revealed that IDH1, IDH2, and PLCH2 are the major driver genes for glioma, clearly suggesting that they may be potential targets for mRNA vaccines.

The high expression of ICPs in IS1 in the TCGA and CGGA cohorts suggested the presence of an immunosuppressive TME, which might suppress the induction of an efficient immune response to mRNA vaccines. Conversely, the elevated expression of ICD regulators in the IS3 and IS4 tumors in the TCGA and CGGA cohorts may be stronger potential for mRNA vaccine application in these immune subtypes. Thus, the complicated immune landscape of glioma shows significant heterogeneity between individuals and within the same immune subtype, which limits the immunogenic target pool for the development of personalized mRNA vaccine-based therapies. This integrated genomic- and genetic-based categorization of glioma should provide the basis for an enhanced molecular understanding of glioma mRNA vaccines that could ultimately result in individualized treatment for groups of patients with glioma. Besides, GP2 and IFNA16 were identified as central genes in the magenta and turquoise modules, and their upregulation was negatively correlated with the immune landscape component, implying that patients expressing these genes at high levels may not respond to mRNA vaccines.

Because mRNA vaccines are closely related to immune status, we further assessed the immune cell compositions of the distinct subtypes. IS1 and IS4 had considerably higher scores for eosinophils, activated CD8 T cells, activated B cells, monocytes, and effector memory CD4 T cells than IS2 and IS3, while memory B cells, macrophages, and memory CD4 T cells had higher scores in IS2 and IS3 than in IS1 and IS4. This result suggests that IS1 and IS4 show immune “active” phenotypes and IS2 and IS3 show immune “suppressive” phenotypes. The molecular characteristics of these tumors were similar to the immune profiles, showing that patients with different immune subtypes have significantly distinguished specificity for mRNA vaccines. Finally, we analyzed the expression of HLA molecules in the immune subgroups, and the expression of HLA molecules in IS1 and IS4 was significantly higher than that in IS2 and IS3, which showed that IS1, IS3, and IS4 may be more sensitive to vaccines than IS2. According to previous immunophenotyping studies of across cancers, glioma was classified into C1-C6 subtypes. C3 was shown to have a better prognosis, C1, C2, and C5 were shown to have a moderate prognosis, and C4 and C6 were shown to have the worst prognosis. In this research, glioma was classified into IS1-IS4 subtypes. IS1 predominantly overlapped with C1, C5, and C6, IS2 predominantly overlapped with C2 and C5, IS3 predominantly overlapped with C1, C2, and C5, and IS4 predominantly overlapped with C1, C3, and C5. Thus, our immune subtypes are reliable and complement the classification schemes previously developed. However, the vaccine antigens and other prognostic markers identified in this study need to be validated in future studies. Finally, **Figure 9** shows the application pattern of mRNA vaccines, which will not be useful for future clinical applications of mRNA.

## Conclusions

TP53, IDH1, C3, and TCF12 are potential glioma antigens for the development of mRNA vaccines. In conclusion, we found four stable and repeatable immune subtypes of human glioma. These subtypes were associated with prognosis, genetic, and immune modulatory alterations that may contribute to the specific types of immune environments we have observed. With our growing knowledge that the tumor immune environment plays an crucial role in prognosis and response to therapy, the classification of the immune subtypes of glioma may play a crucial role in the predicting mRNA vaccine outcome.

## Data Availability Statement

The original contributions presented in the study are included in the article/**Supplementary Material**. Further inquiries can be directed to the corresponding authors.

## Ethics Statement

Written informed consent was obtained from the individual(s) for the publication of any potentially identifiable images or data included in this article.

## Author Contributions

SM, JD, and SH conceived and designed the study and drafted the manuscript. SM, YB, HJ, and FW provided analytical technical support. All authors contributed to the article and approved the submitted version.

## Funding

This work was funded by the National Natural Science Foundation of China (No. 61575058).

## Conflict of Interest

The authors declare that the research was conducted in the absence of any commercial or financial relationships that could be construed as a potential conflict of interest.

## Publisher’s Note

All claims expressed in this article are solely those of the authors and do not necessarily represent those of their affiliated organizations, or those of the publisher, the editors and the reviewers. Any product that may be evaluated in this article, or claim that may be made by its manufacturer, is not guaranteed or endorsed by the publisher.
