# Supplementary material for: Recognition of Tumor-Associated Antigens and Immune Subtypes in Glioma for mRNA Vaccine Development
Source: Front Immunol. 2021 Sep 17;12:738435. doi: 10.3389/fimmu.2021.738435 (PMC8484904; doi:10.3389/fimmu.2021.738435)
Supplement: Supplementary file 1 [file DataSheet_1.zip › Data_Sheet_1.docx]

# Supplementary Material

## Supplementary Figures


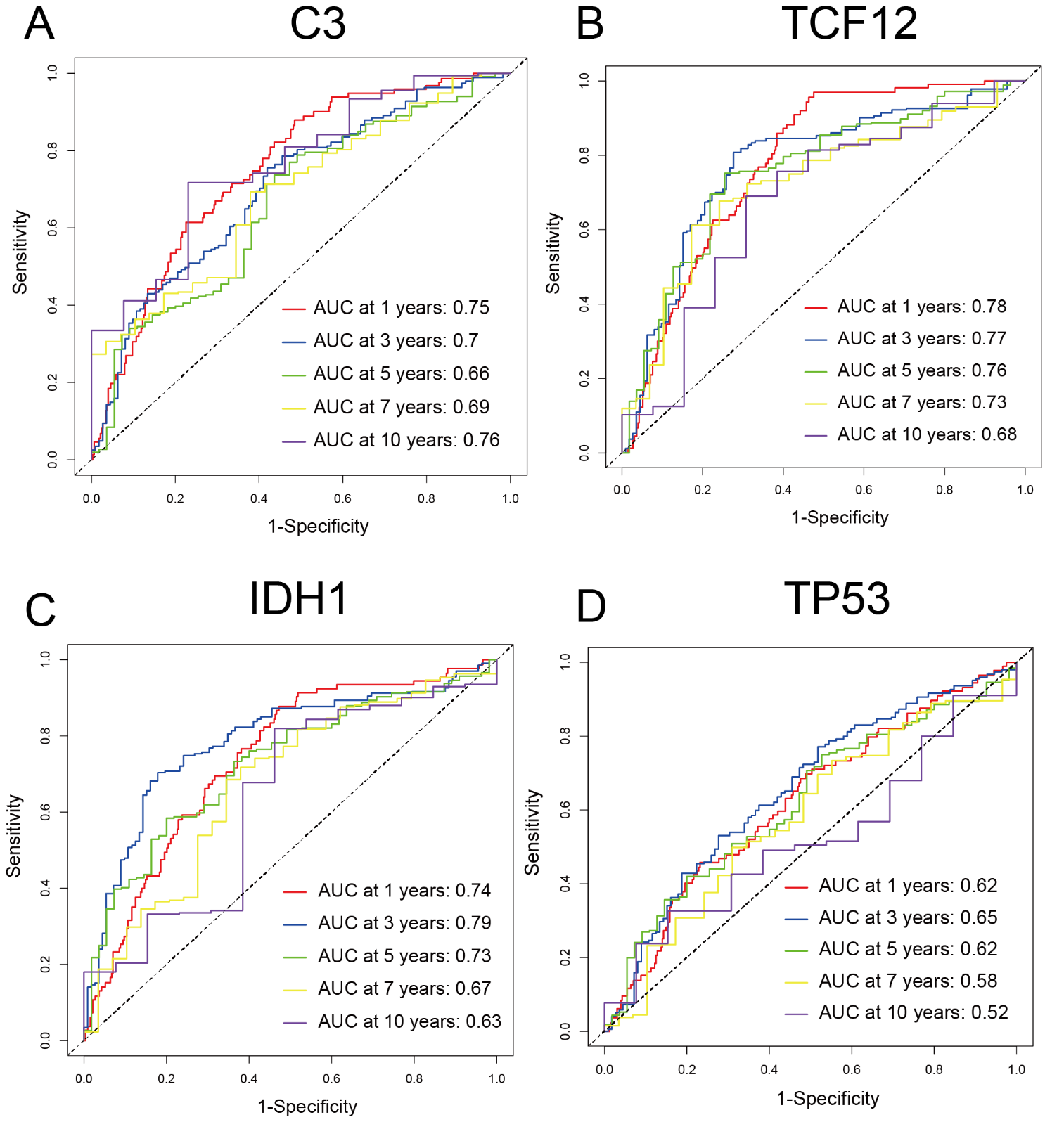


**Figure S1 The ROC of 4hub genes in TCGA cohort.**

**(A-D)** Validation of 1-year, 3-year, 5-year, 7-year, 10-year ROC curves for 4 hub genes.


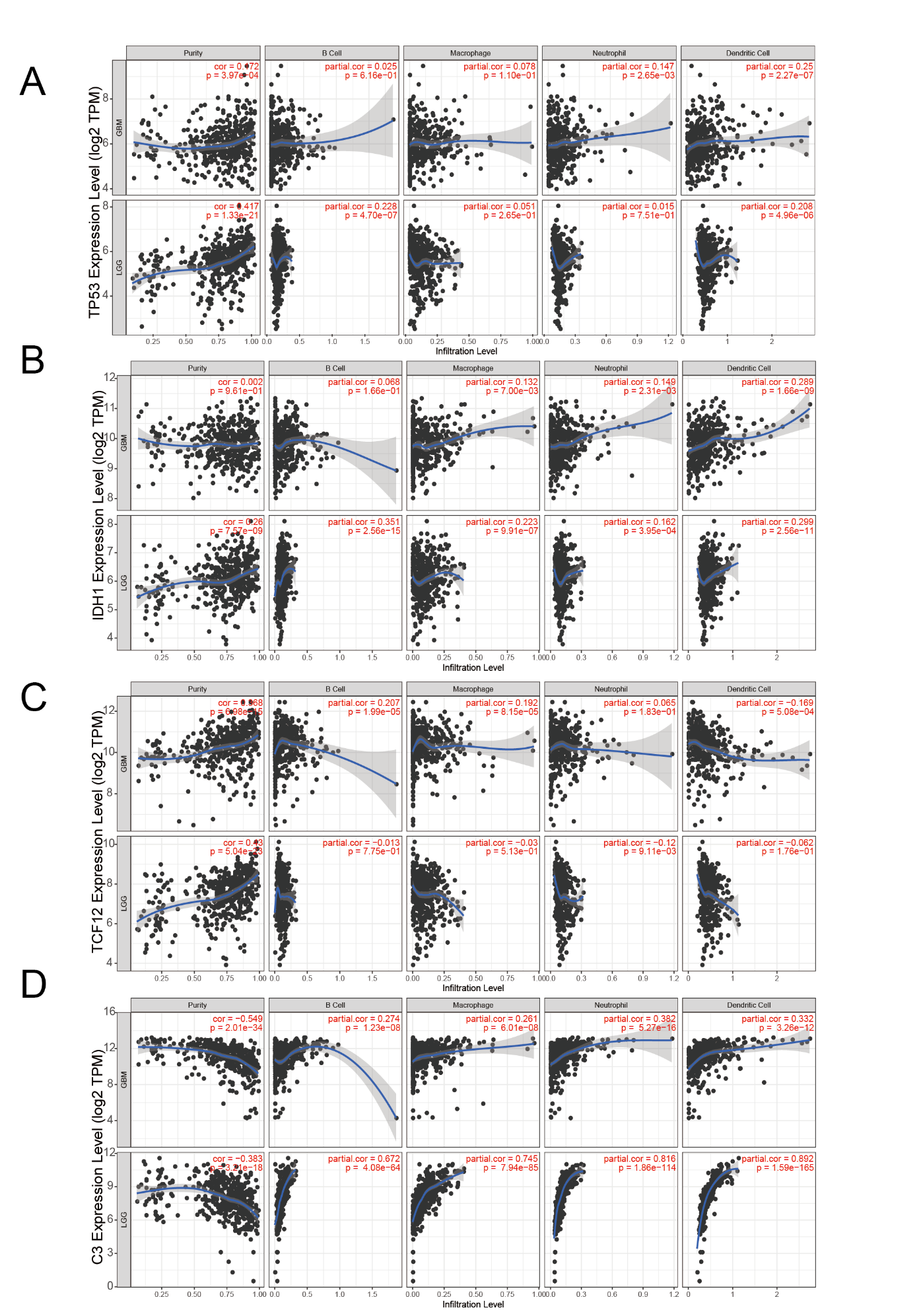


**Figure S2.** **Identification of APC-related tumor antigens.**

(A-D) The relevance among the expression levels of (A) TP53, (B) IDH1, (C) TCF12, (D) C3.


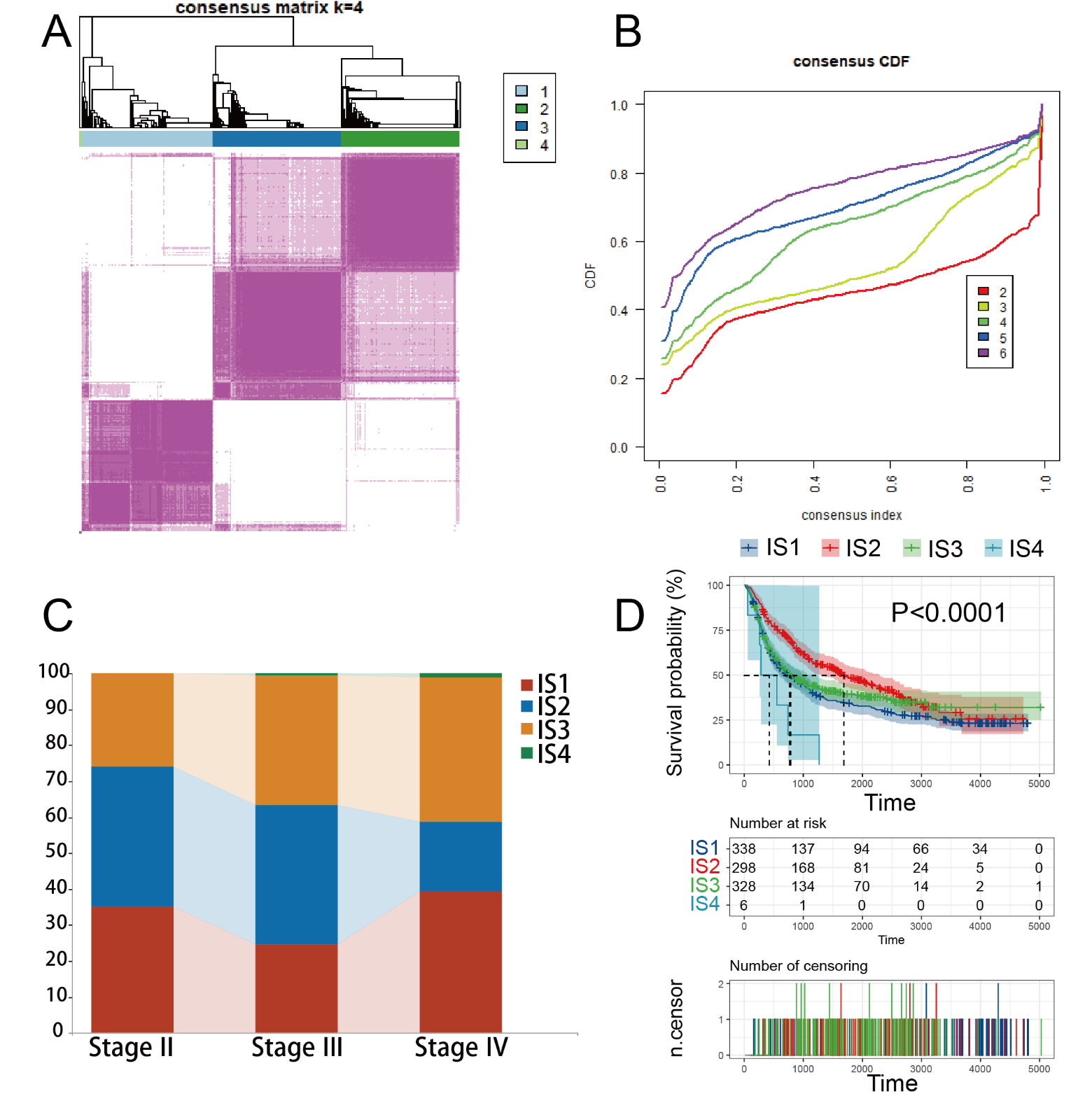


**Figure S3. Consensus cluster in CCGA.**

**(A)** Color-coded heat maps corresponding to the consensus matrices. **(B)** Consensus Cumulative Distribution Function (CDF) Plot for immune-related genes for glioma. **(C)** Distribution of IS1-IS4 between WHO stages in the CCGA cohort. **(D)** Kaplan-Meier curves plot over survival of glioma 4 immune subtypes in CCGA cohort.


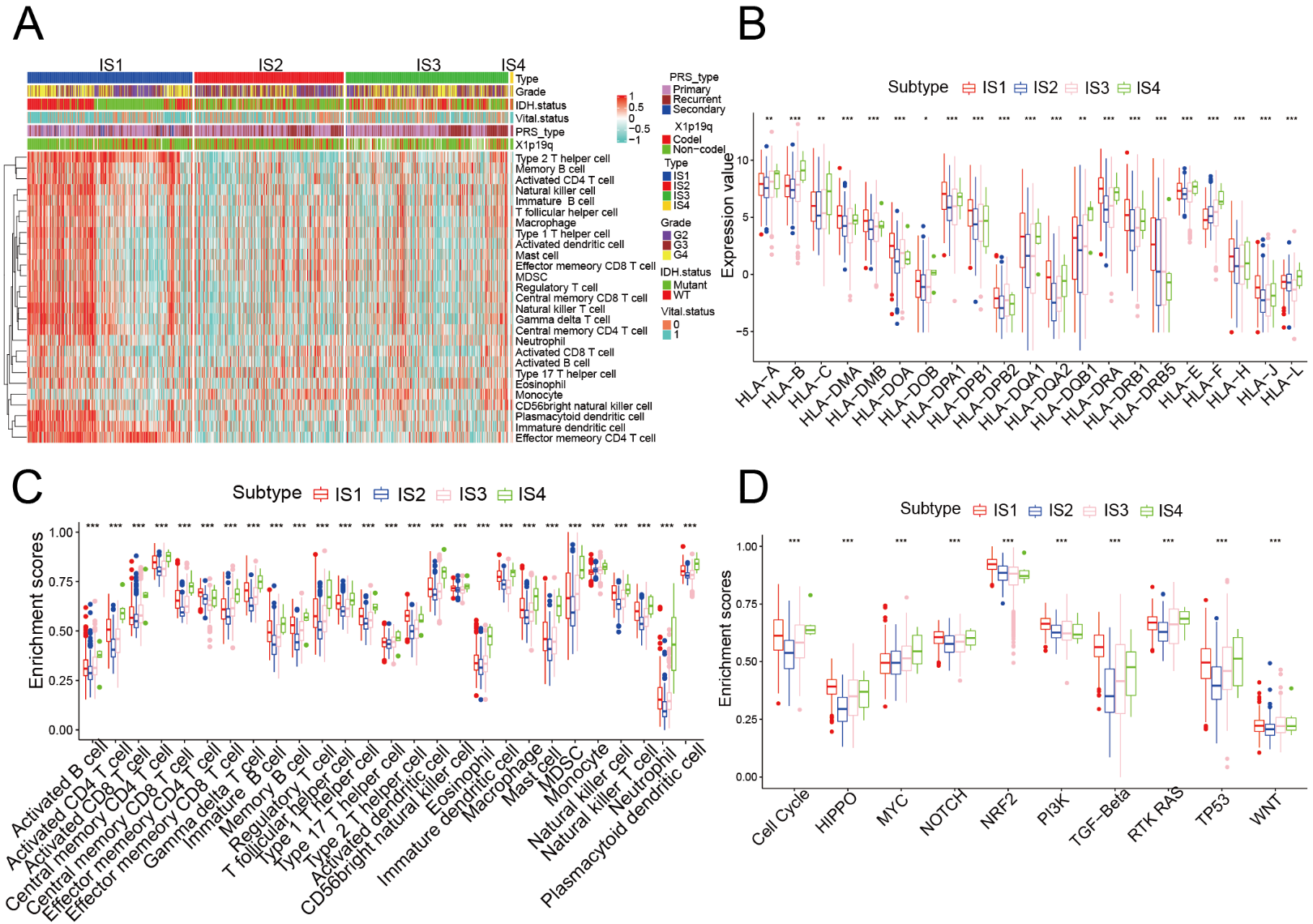


**Figure S4.** **Immune types of immune cells infiltration in CCGA.**

**(A)** Heatmap analysis of the distinct enrichment scores of 28 immune cell features among glioma immune subtypes. **(B-D)** Quantitative analysis of the distribution of *HLA* (b), 28 immune cells (c), and 10 oncogenic pathways in the immune types (d).


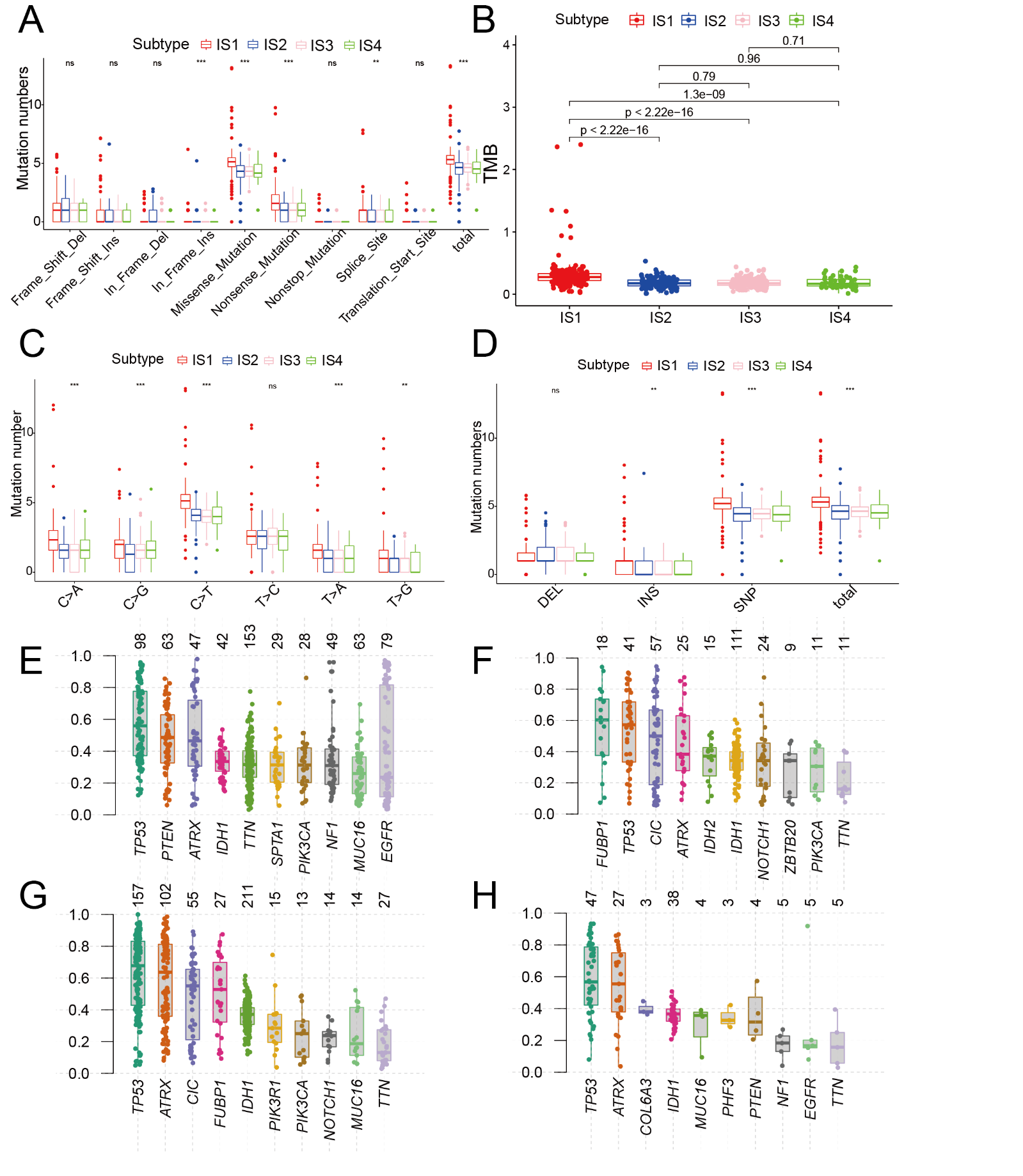


**Figure S5.** **Distribution of mutations at the gene level among immune subgroups.**

**(A)** Each mutation type is classified by effects, INDEL, and SNV (c), SNP (d). **(B)** TMB distribution of 4 immune types in TCGA. **(E-H)** Variant Allele Frequency expression of the top 10 genes of the four immune subgroups.


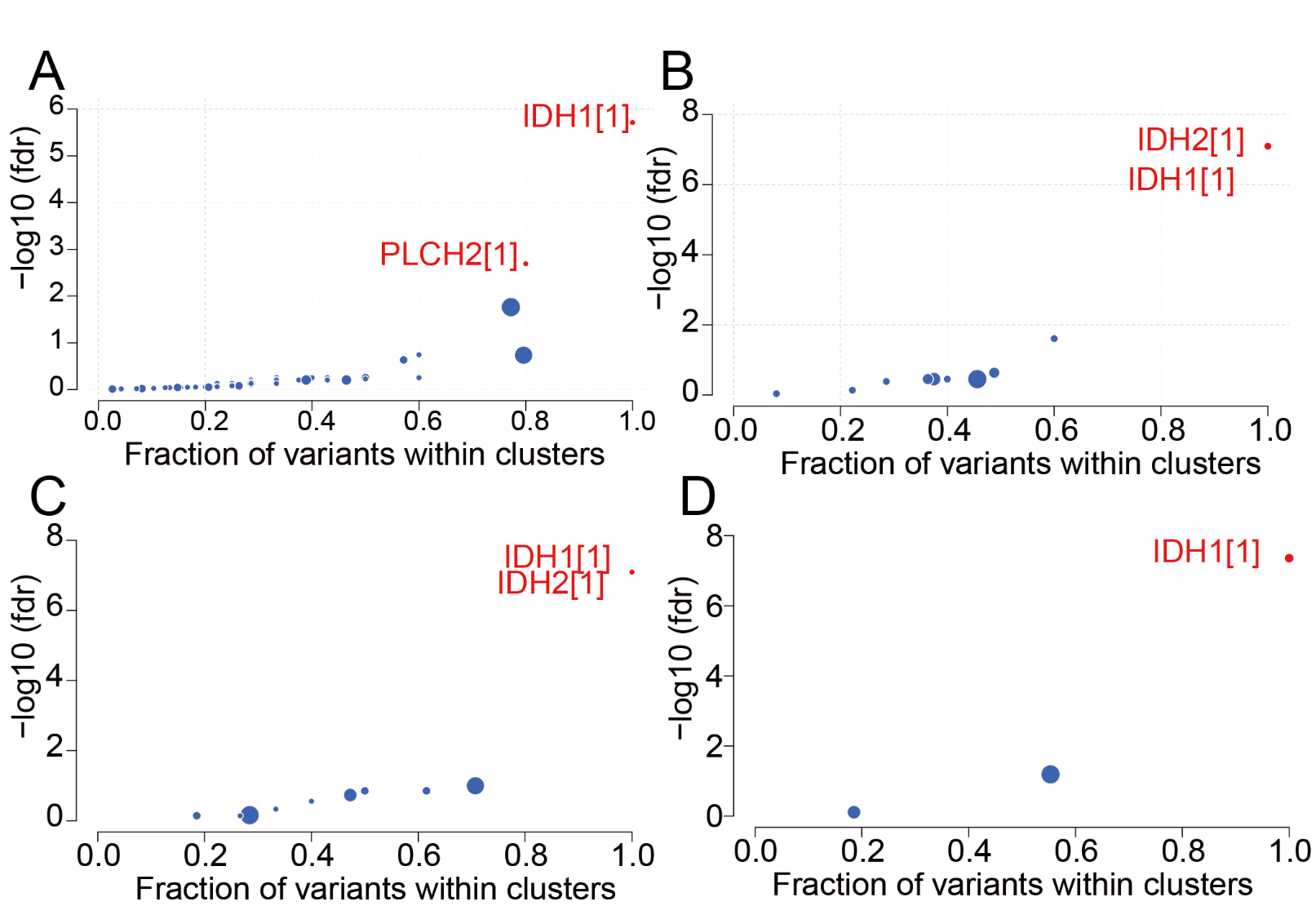


**Figure S6.** Identification of tumor driver genes for 4 immune subtypes.

**(A-D)** Distribution of tumor driver genes in IS1 (a), IS2 (b), IS3 (c), IS4 (d).


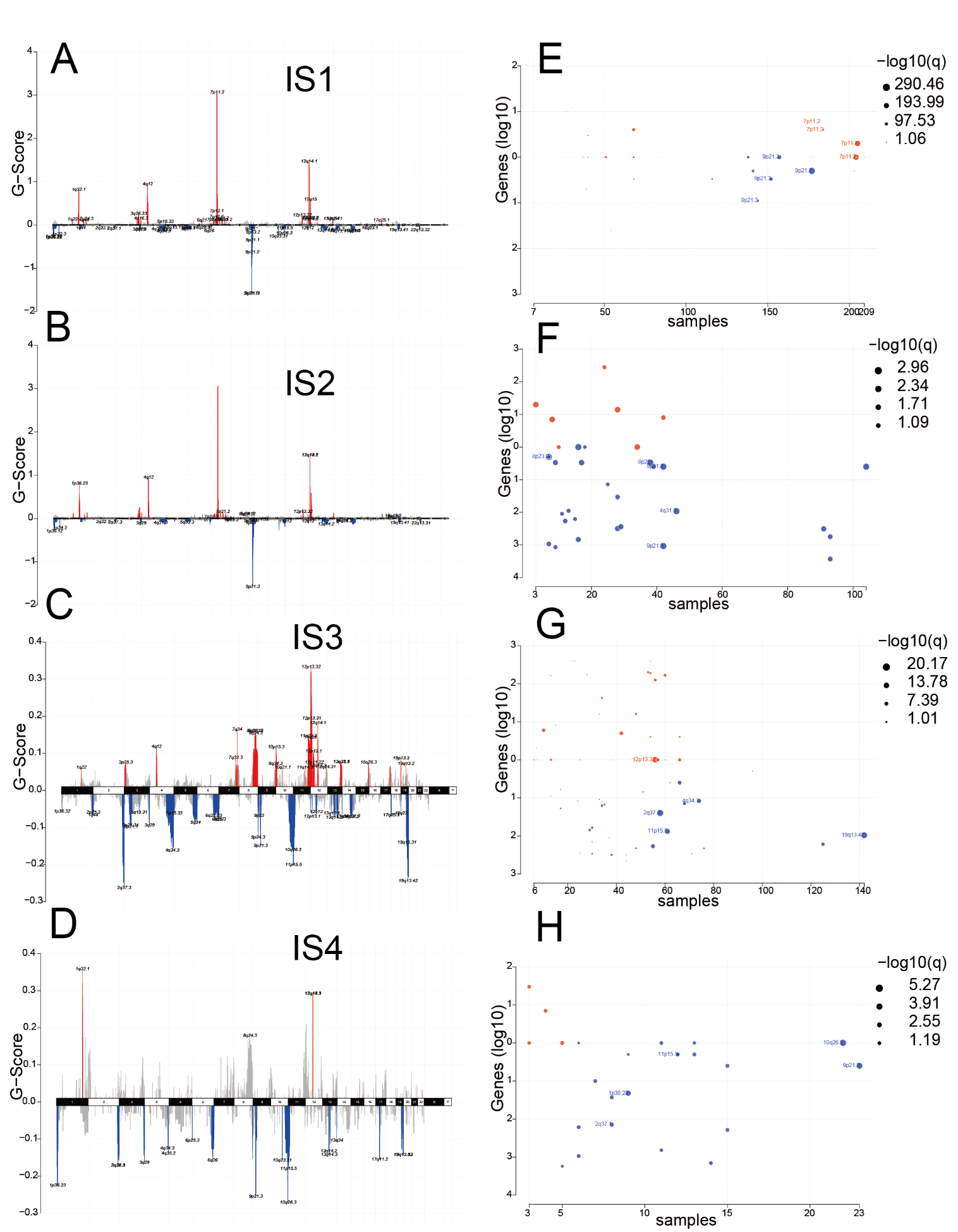


**Figure S7. Visualization of 4 immune subtypes of CNV.**

**(A-D)** Significant amplifications and deletions of G-score were detected among 4 immune types. **(E-H)** Distribution of Amp and Del in four immune subtypes.


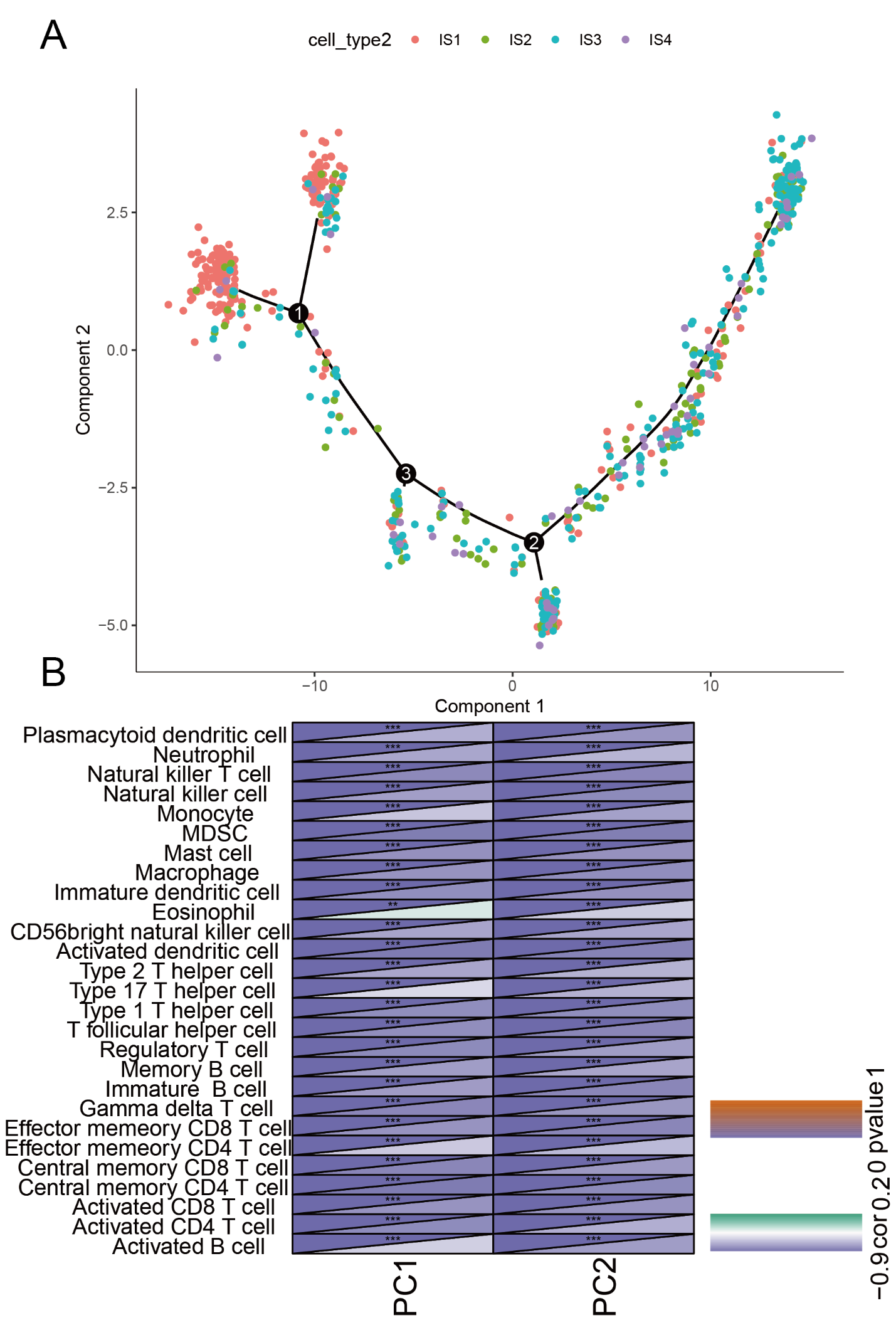


**Figure S8. The immune landscape of glioma.**

**(A)** A patient is represented by each dot and immune subtypes are encoded by color. The horizontal coordinates indicate the first primary component and the vertical coordinates indicate the second primary component. **(B)** Heatmap of both primary components with characteristics of 28 immune cells.


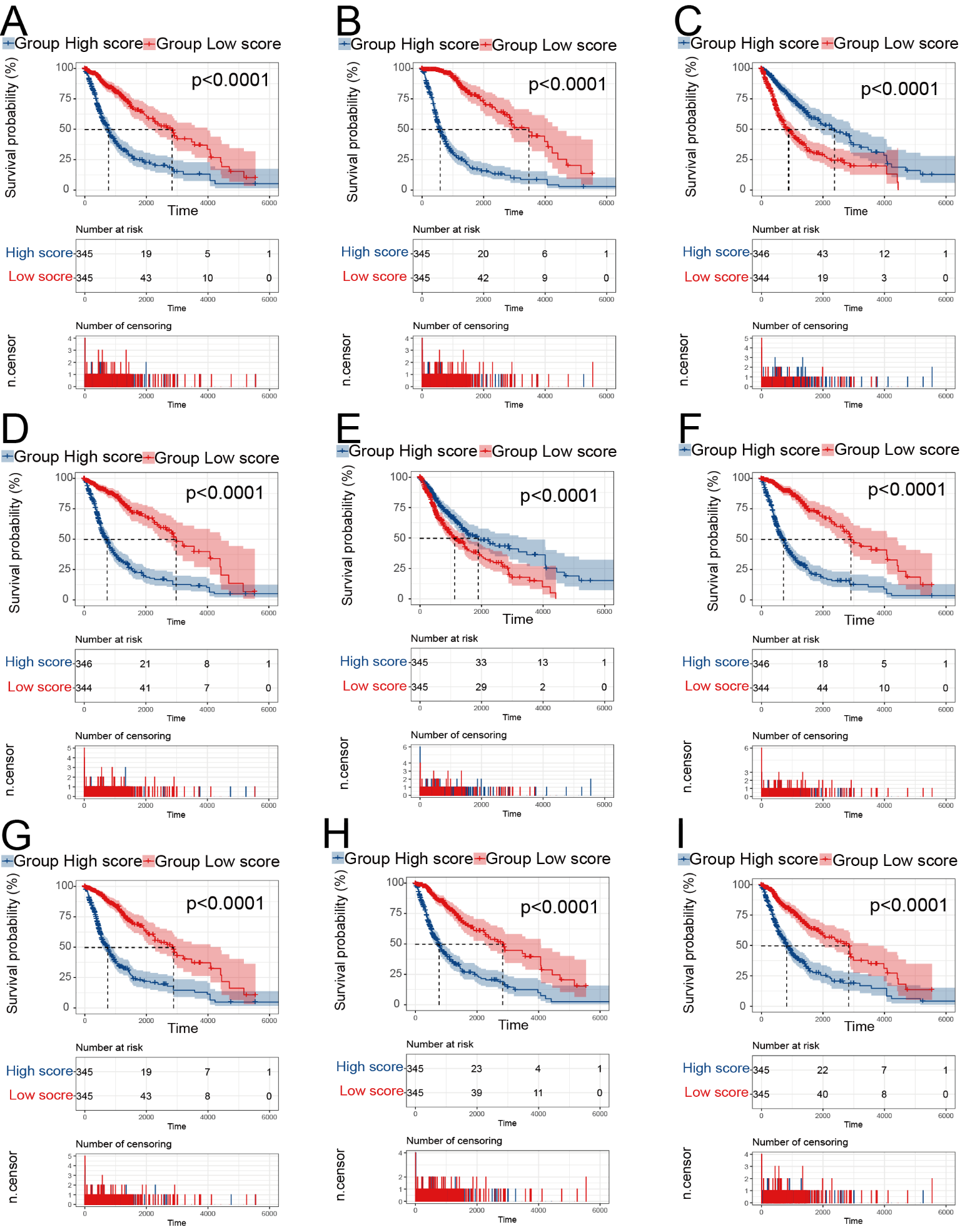


**Figure S9. Kaplan-Meier curves for 9 modules.**

**(A-I)** Median values were taken and divided into high and low scoring groups for survival analysis of the 9 modules.


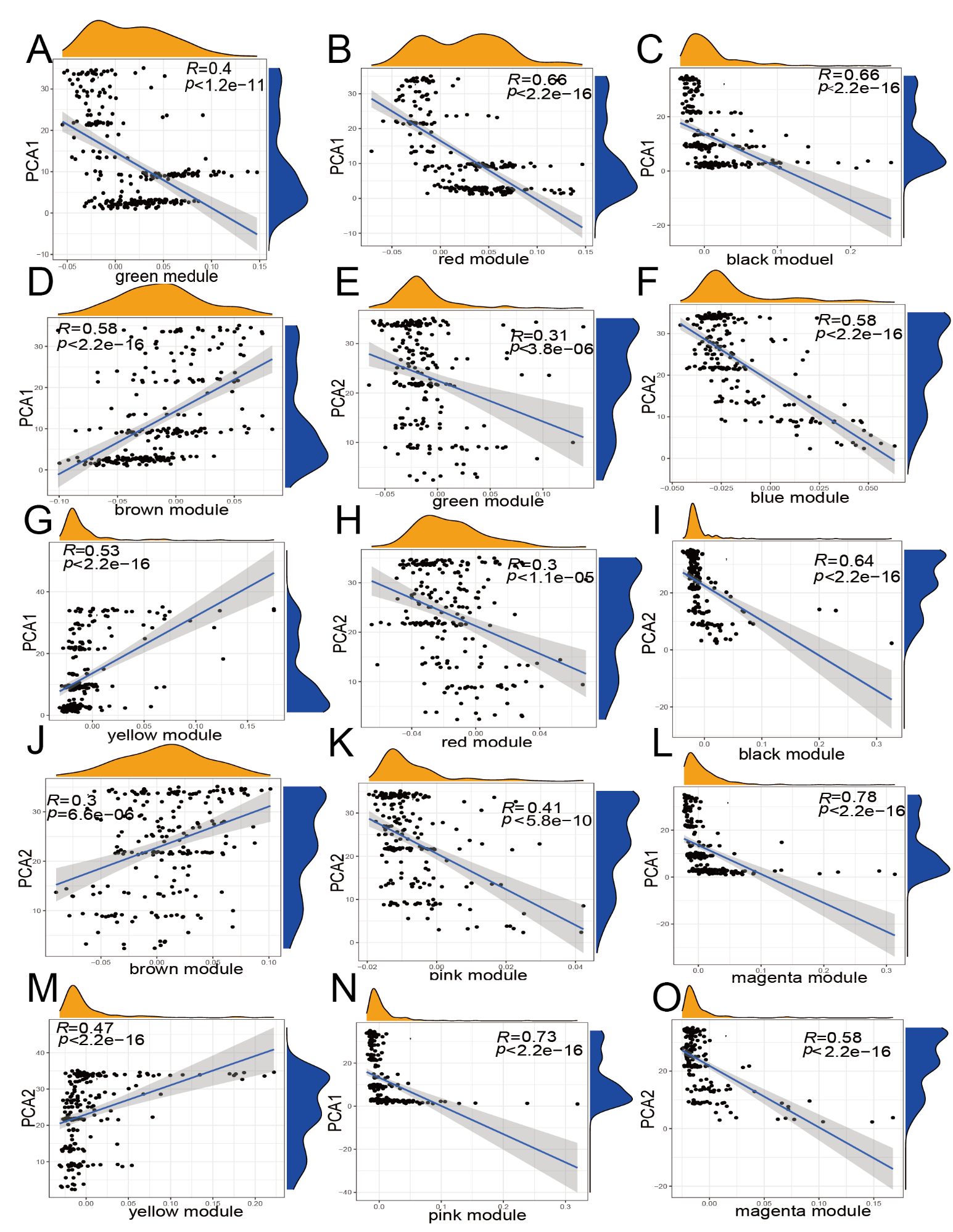


**Figure S10. Correlation analysis of two principal components with nine modules.**

**(A-G)** Correlation analysis of the first principal component with the nine modules. **(H-O)** Correlation analysis of the second principal component with the nine modules.


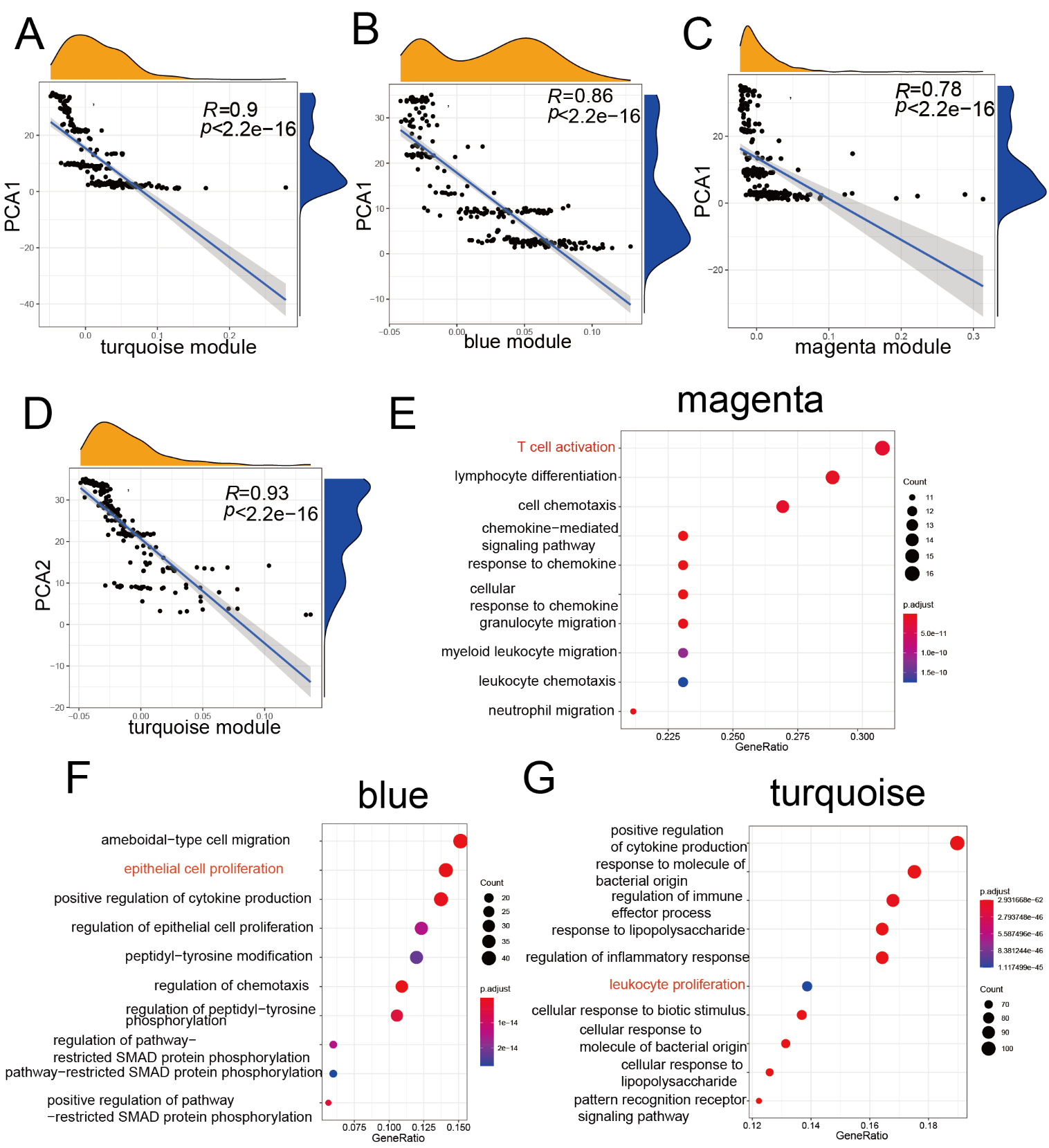


**Figure S11 Identification of glioma immune key genes.**

**(A-D)** Relevance between turquoise, blue, and magenta modules eigenvectors and two primary components in immune landscape. **(E-G)** The magenta, blue, turquoise module genes is displayed the top 10 terms by GO enrichment analysis. The size and color intensity of the points represent the number of genes and the enrichment, respectively.


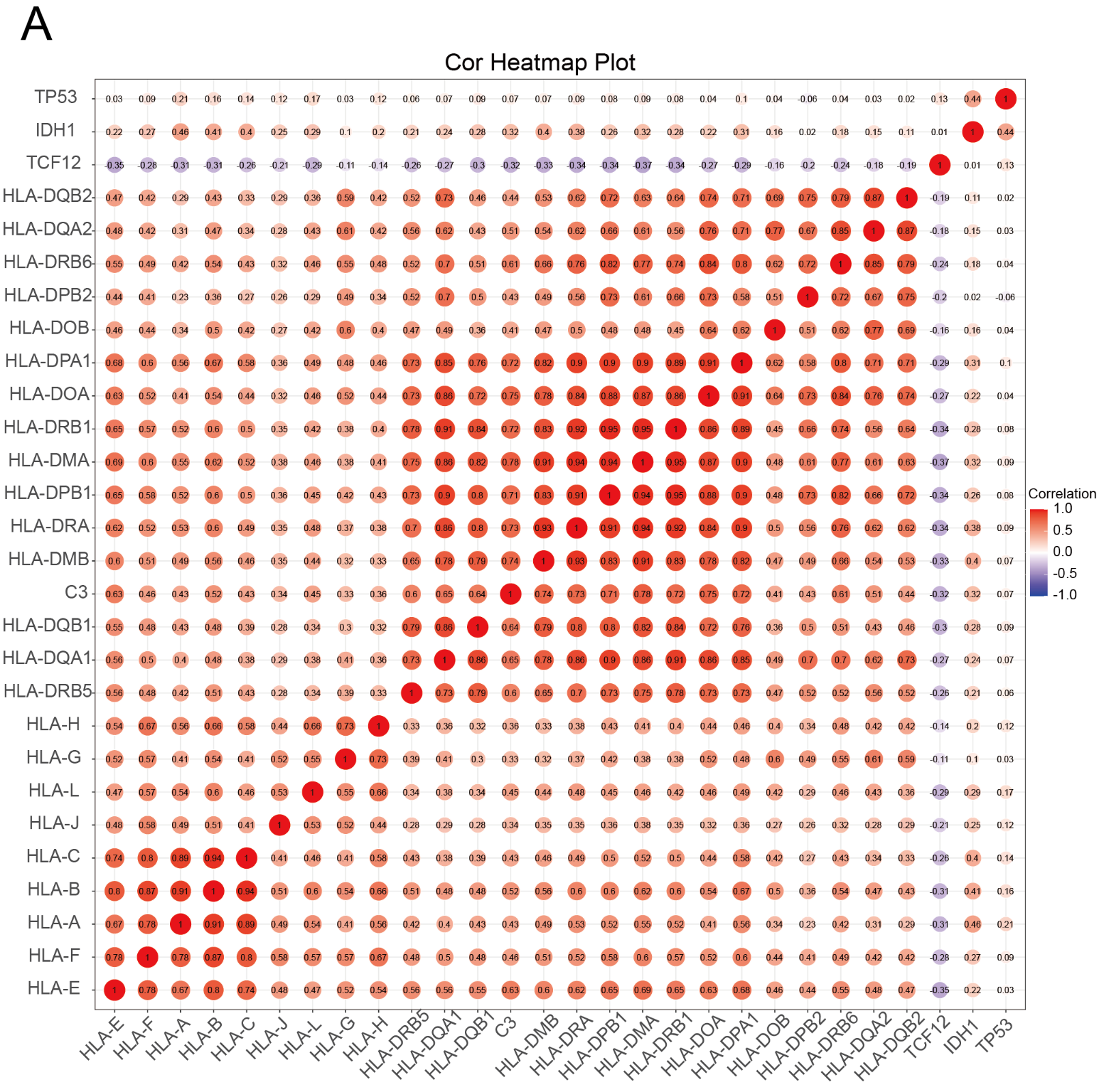


**Figure S12 The Correlation of 4hub genes with HLA antigens.**

**(A)** The Correlation of 4 hub genes with HLA antigens.
